# Identification of differentially expressed long noncoding RNAs and pathways in liver tissues from rats with hepatic fibrosis

**DOI:** 10.1371/journal.pone.0258194

**Published:** 2021-10-01

**Authors:** Yan Wang, Xiong Xiao, Xiaobo Wang, Feng Guo, Xiaozhong Wang

**Affiliations:** 1 Department of Traditional Chinese Medicine, The Fifth People’s Hospital Affiliated to Chengdu University of Traditional Chinese Medicine, Chengdu, China; 2 Department of Liver Disease, Traditional Chinese Medicine Hospital Affiliated to Xinjiang Medical University, Urumqi, China; Amity University Haryana, INDIA

## Abstract

To identify long non-coding RNAs (lncRNAs) and their potential roles in hepatic fibrosis in rat liver issues induced by CCl4, lncRNAs and genes were analyzed in fibrotic rat liver tissues by RNA sequencing and verified by quantitative reverse transcription polymerase chain reaction (qRT-PCR). Differentially expressed (DE) lncRNAs (DE-lncRNAs) and genes were subjected to bioinformatics analysis and used to construct a co-expression network. We identified 10 novel DE-lncRNAs that were downregulated during the hepatic fibrosis process. The cis target gene of DE-lncRNA, XLOC118358, was *Met*, and the cis target gene of the other nine DE-lncRNAs, XLOC004600, XLOC004605, XLOC004610, XLOC004611, XLOC004568, XLOC004580 XLOC004598, XLOC004601, and XLOC004602 was *Nox4*. The results of construction of a pathway-DEG co-expression network show that lncRNA-Met and lncRNAs-Nox4 were involved in oxidation-reduction processes and PI3K/Akt signaling pathway. Our results identified 10 DE-lncRNAs related to hepatic fibrosis, and the potential roles of DE-lncRNAs and target genes in hepatic fibrosis might provide new therapeutic strategies for hepatic fibrosis.

## Introduction

Hepatic fibrosis results from liver tissue repair disorder after injury, and is characterized by pathological and excessive deposition of extracellular matrix (ECM) [1–3]. Hepatic stellate cells (HSCs) are activated and transformed into myofibroblast-like cells, which secrete a large amount of ECM, including α smooth muscle actin (αSMA) and type I collagen alpha 1 (ColIα1) [4]. Transforming growth factor β 1 (TGFβ1) is the main pro-fibrosis factor, and the TGF-β/Smad signaling pathway is the most important intracellular signaling pathway in the occurrence and development of liver fibrosis [5,6].

Long noncoding RNA (lncRNA) is endogenous RNA with a molecular weight of more than 200 nucleotides. It exists in the cytoplasm or nucleus and lacks an effective open reading frame and protein coding function. Numerous studies have shown that lncRNAs play an important role in the formation and development of liver fibrosis, mainly through transcriptional regulation [7], increasing the stability of mRNA [8], reducing the stability of mRNA [9], promoting mRNA translation [10], inhibiting mRNA translation [11], ceRNA mechanism [12], and regulation of the precursor of microRNA [13]. In the nucleus, lncRNAs can not only regulate gene transcription through various mechanisms, but also regulate at the post-transcriptional level, such as in the splicing of mRNA precursors [14].

In recent decades, several studies on liver fibrosis-related diseases have shown the upregulated expression of lncRNAs in liver fibrosis included MALAT1 [13,15], APTR [16], and lncRNA-ATB [17]. In contrast, lincRNA-p21 [11], Meg3, Gas5 [18,19], H19 [20,21], and HIFIA-AS1 [22,23] were shown to be downregulated. In addition, MALAT1 [24], APTR [16], HULC [25] and Hox-antisense-RNA [26] were found to be upregulated in liver cirrhosis [13]. In contrast, lincRNA-p21 [11] and Meg3 [27] were downregulated. These lncRNAs may be potential therapeutic targets for liver fibrosis and liver cirrhosis [21]. Furthermore, uncovering the mechanism behind the interaction between lncRNA-miRNA/mRNA may provide a new strategy for the treatment of liver fibrosis-related diseases [28].

However, the number of lncRNA families is very large, and further research is needed to reveal whether there are other lncRNAs that play a role in the process of liver fibrosis. Therefore, in this study, we performed high-throughput sequencing of RNA in liver tissues from rats with CCl4-induced liver fibrosis. Co-expression networks were constructed to explore regulatory lncRNAs and their targets. Gene Ontology (GO) terms and KEGG pathways analysis technologies were used to identify the functions of several lncRNAs associated with hepatic fibrosis. The results were verified by quantitative polymerase chain reaction (qRT-PCR).

## Materials and methods

### Establishment of CCl4 induced hepatic fibrosis in rats

SPF male SD rats (Certificate No. scxk (New) 2016–0003) with a body weight of 200 ± 20 g were purchased from the Animal Experimental Center of Xinjiang Medical University, and the certificate number of experimental animals was 650007000981. Twenty-five rats were randomly divided into two groups: normal control group (n = 10) and hepatic fibrosis group (n = 15). Rats in the liver fibrosis group (FL) were intraperitoneally injected with CCI4 (CCI4: olive oil = 1:1, 2 mL/kg body weight, twice a week) for five weeks, and the control group (CL) was administered the same volume of olive oil. Animals were observed daily and euthanized when they appeared to lose 15–20% of their original body weight rapidly, or growing animals showed no weight gain or lost appetite chronically. Experiments were terminated when significant differences in animal metrics between groups were observed, reducing unnecessary animal suffering. After treatment with CCl4 for five weeks, all rats were sacrificed under anesthesia with 3% sodium pentobarbital (35 mg/kg). Liver tissues of the rats in both groups were stained with hematoxylin-eosin (HE), immunohistochemistry (α-SMA and col1α1), and hydroxyproline kit to determine the hydroxyproline content. The experiment was carried out with the approval of the Ethics Committee of the Xinjiang Uygur Autonomous Region Medical Research Institute (nG4-2019012).

### RNA isolation, library preparation, and sequencing analysis

TRIzol (Invitrogen, Carlsbad, CA, USA) was used to extract total RNA from rat liver tissues and the extracted RNA was purified according to the manufacturer’s instructions. A NanoDrop ND-2000 spectrophotometer (Thermo Fisher Scientific, Waltham, MA, USA) was used to quantify total RNA, and RNA integrity was determined using an Agilent 2100 system and RNA 6000 Nano Kit (Agilent Technologies, Santa Clara, CA, USA). To achieve accurate lncRNA analysis, ribosomal RNA was removed using the Thermo Fisher Scientific Central Ribonucleic Acid Removal Kit. Next-generation sequencing (NGS) analysis was performed using NGS technology on an Illumina Genome Analyzer IIx (Illumina, San Diego, CA, USA).

### RNA-Seq raw data clean and alignment

The original reads containing more than 2-N bases were discarded. Short reads of less than 16nt, adapters, and low-quality bases in the original sequencing reads were removed using the FASTX-Toolkit (Version 0.0.13). Clean reads were then compared with the GRCH38 genome using TopHat2 [29] allowing four mismatches. Unique locational reads were used for gene read count and FPKM (number of locational fragments per million segments per 1,000 base transcripts) [30].

### Differentially expressed gene (DEG) analysis

The R Bioconductor package edgeR [31] was used to locate the differentially expressed genes (DEGs). A selected threshold, false discovery rate <0.05 and fold change>2 or < 0.5, was set as the cut-off criteria for identifying DEGs.

### LncRNA prediction

The lncRNA prediction pipeline followed the method described in a previous study [32] as follows:

1. According to the RNA-seq comparison results, the default parameters were used to assemble the transcript using Cufflinks V2.2 [33]. After the initial assembly, transcripts not less than 0.3 of FPKM were retained for subsequent filtration.
2. Embed Cuffcompare in Cufflinks, transcripts were compared with known internal reference genome genes, and new transcripts, such as intergenic, intron, and antisense regions, were reserved as candidate lncRNAs. Transcripts within 1,000 bp that were adjacent to the known encoding genes were considered UTRs and were also discarded.
3. The coding potential score (CPS) was evaluated using the coding potential calculator (CPC) software [34], and the coding potential report card was screened. CPC is a support vector machine-based classifier that evaluates the protein-coding potential of transcripts based on six sequence features of biological significance. Transcripts with a CPS below 0 were considered non-coding RNAs.
4. For transcripts meeting the above conditions, multiple exons no smaller than 200 bases and single exons no smaller than 1,000 bases were reserved as lncRNAs.
5. Finally, the known and predicted lncRNAs in all samples were combined to obtain the final set of lncRNAs, and the expression level of each lncRNA gene was recalculated. The antisense reading of lncRNAs was discarded.

### Differentially expressed (DE) lncRNAs

After the expression levels of all lncRNAs in all samples were obtained, edgeR [31], one of the R packages, was used to analyze the DE-lncRNAs. For each lncRNA, the *P*-value was obtained using a negative binomial distribution model. Folding changes were also estimated using this package. The threshold values of DE-lncRNAs were defined as 0.05 q-value of two times change.

### Cis acting targets

Correlation coefficients and *P*-values were obtained for each mRNA-DE-lncRNA pair based on the expression of each mRNA and DE-lncRNA. We then filtered the result by a given threshold, with an absolute correlation coefficient of no less than 0.6 and a *P*-value less than 0.05. In addition to the positive correlation pairs, negative pairs with correlation coefficients less than 0 were also included. For each DE-lncRNA, we obtained expressed genes from its upstream and downstream regions within 10,000 bases, and these genes were overlapped with co-expressed genes to obtain lncRNA targets.

### Functional enrichment analysis

DEGs were classified functionally using KOBAS 2.0 server [35] with Gene Ontology (GO) functional enrichment analysis and Kyoto Encyclopedia of Genes and Genomes (KEGG) analysis. The enrichment of all items was defined by the hypergeometric test and the Benjamini-Hochberg FDR control method.

### One-step reverse transcription quantitative polymerase chain reaction(qRT-PCR) validation

The DE-lncRNA and co-expression mRNA provided by RNA-seq were reverse transcribed into cDNA using the one-step RT method, and then primers were designed according to the results provided by gene sequencing analysis, and qPCR amplification was performed. The expression of DE-lncRNA and co-expression mRNA was detected in the FL and CL groups. qPCR analysis was performed using the same three biological replicates as RNA-seq.

### Construction of a pathway-DEG co-expression network

The top10 GO P terms/TOP 10 KEGG terms and their corresponding genes were extracted from DEG, and the network was drawn using Cytoscape software to show the relationship between genes and pathways.

### Availability of data and materials

The data discussed in this publication are available under GEO Series accession number GSE173085.

### Statistical analysis

IBM SPSS version 26.0 was used for the analysis. All data were expressed as mean ± standard deviation, and Student’s t-test was used for the comparison between groups. Differences were considered statistically significant at *p* < 0.05, between the control group and the model group, and GraphPad Prism 8.0 was used for drawing.

## Results

### Establishment of CCl4-induced hepatic fibrosis in rats

The results of macroscopic examination in S1 Fig illustrate that the liver surface of the CL group was smooth, and the nodules on the surface of the FL group were obvious (S1A Fig).

HE staining showed that the structures of hepatic lobules in the CL group were normal, and the hepatocytes were arranged radially around the central vein, in an orderly manner. In the FL group, the structure of the liver tissue was disordered, the structure of the hepatic lobule was destroyed, the fibrous septum between lobules was thick, the fibrous connective tissue in the portal area proliferated, and the hepatic lobules were divided and wrapped into pseudo lobules of different sizes and shapes (S1B Fig).

Compared with the CL group, the degree of fibrosis in the FL group was significantly higher, according to the Ishak score of liver fibrosis (S1D Fig).

As shown in S1C Fig, immunohistochemical staining results showed that α-SMA and colIα1 proteins were mainly expressed in the cytoplasm, and DAB staining was brown yellow. In the CL group, α-SMA and colIα1 were negative. In the FL group, the number of positive cells for α-SMA and colIα1 increased significantly, and a large number of brown yellow staining was found in the fibrous septum and hepatic sinuses.

Real-time fluorescence quantitative PCR results showed that α-SMA and colIα1 mRNA levels in liver tissue were significantly increased after CCl4 induction. Hydroxyproline is a non-essential amino acid, almost all of which is found in collagen, except for a small amount in elastin. Collagen is the main component of ECM in liver fibrosis; therefore, the detection of hydroxyproline content in liver tissue directly reflects the degree of liver fibrosis. The hydroxyproline content in the liver tissue increased three to four times after the intraperitoneal injection of CCl4 (S1E Fig).

These results suggested that CCl4 successfully induced liver fibrosis in the rats. Finally, the rats numbered CL5, CL6, and CL7 in the CL group and FL2, FL5, and FL12 in the FL group were included in the RNA-seq test.

### DE-LncRNAs during hepatic fibrogenesis

**Fig 1:**
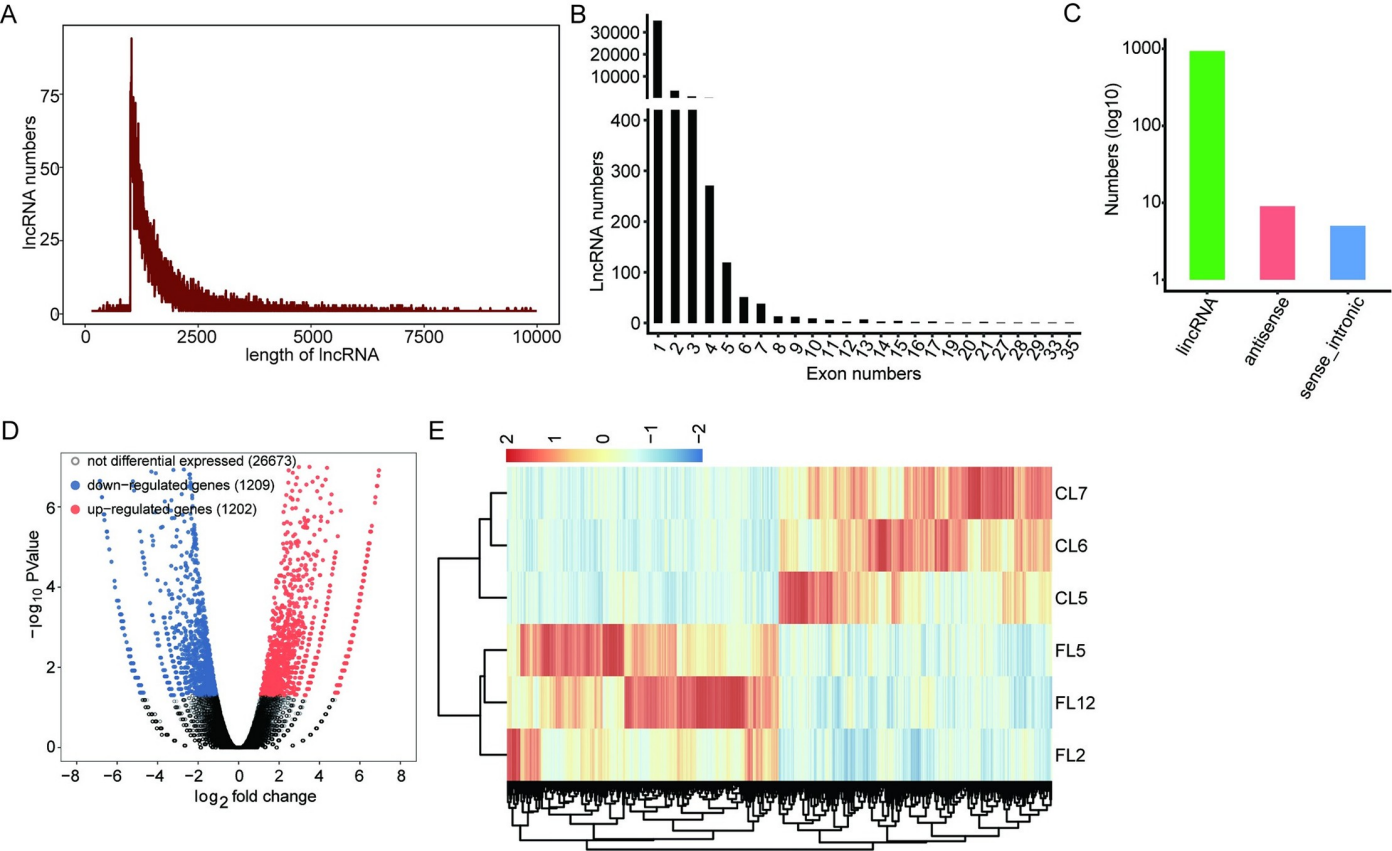
LncRNA expression profile during hepatic fibrogenesis. (A) Distribution of lncRNA length. (B) Statistics of exon number in lncRNA. (C) Classification of lncRNA. (D) Identification of DE-lncRNAs. Upregulated lncRNAs are labeled in red, whereas downregulated are labeled in blue in the volcano plot. FC ≥ 2 or ≤ 0.5, FDR < 0.05. (E) Hierarchical clustering of DE-lncRNAs in control and fibrotic rat liver samples. The color scale represents the median-centered log2 RPKM values.

Total RNA was extracted from three fibrotic liver tissues (FL2, FL5, and FL12) and three normal liver tissues (CL5, CL6, and CL7). After quality inspection, we performed high-throughput transcriptome sequencing. The lncRNA expression profile during hepatic fibrosis is shown in Fig 1. We obtained >89,784,860 clean reads that consisted of raw data from the Illumina HiSeq platform. TopHat was used to map the genome of clean reads, and Cufflinks was used to reconstruct the transcript. After basic filtering, 29,084 lncRNAs were screened. The length of lncRNAs ranged from 200 to 10,000 bases, and the most frequent length was approximately 1,000 to1,500 bases (Fig 1A). The number of lncRNA exons ranged from 1 to 35, and the largest number of lncRNAs with exon numbers ranged from one to three (Fig 1B). The classification of lncRNAs included lincRNA, antisense lncRNA, sense intronic lncRNA, and lincRNA accounted for the highest proportion (Fig 1C). Finally, we found that there were 2,411 DE-lncRNAs between fibrotic liver tissues and normal liver tissues, of which 1,202 were upregulated and 1,209 were downregulated (Fig 1D). A heat map showing the hierarchical clustering of DE-lncRNAs was observed between the control groups and the fibrotic livers. Compared with previous studies, it was found that the length of mRNAs was more than 1,000 bp, and the number of exons was mostly between 3 and 20. Compared with protein-coding genes, lncRNAs have shorter lengths and fewer exons.

### Screen and validation of DE-lncRNAs

**Fig 2:**
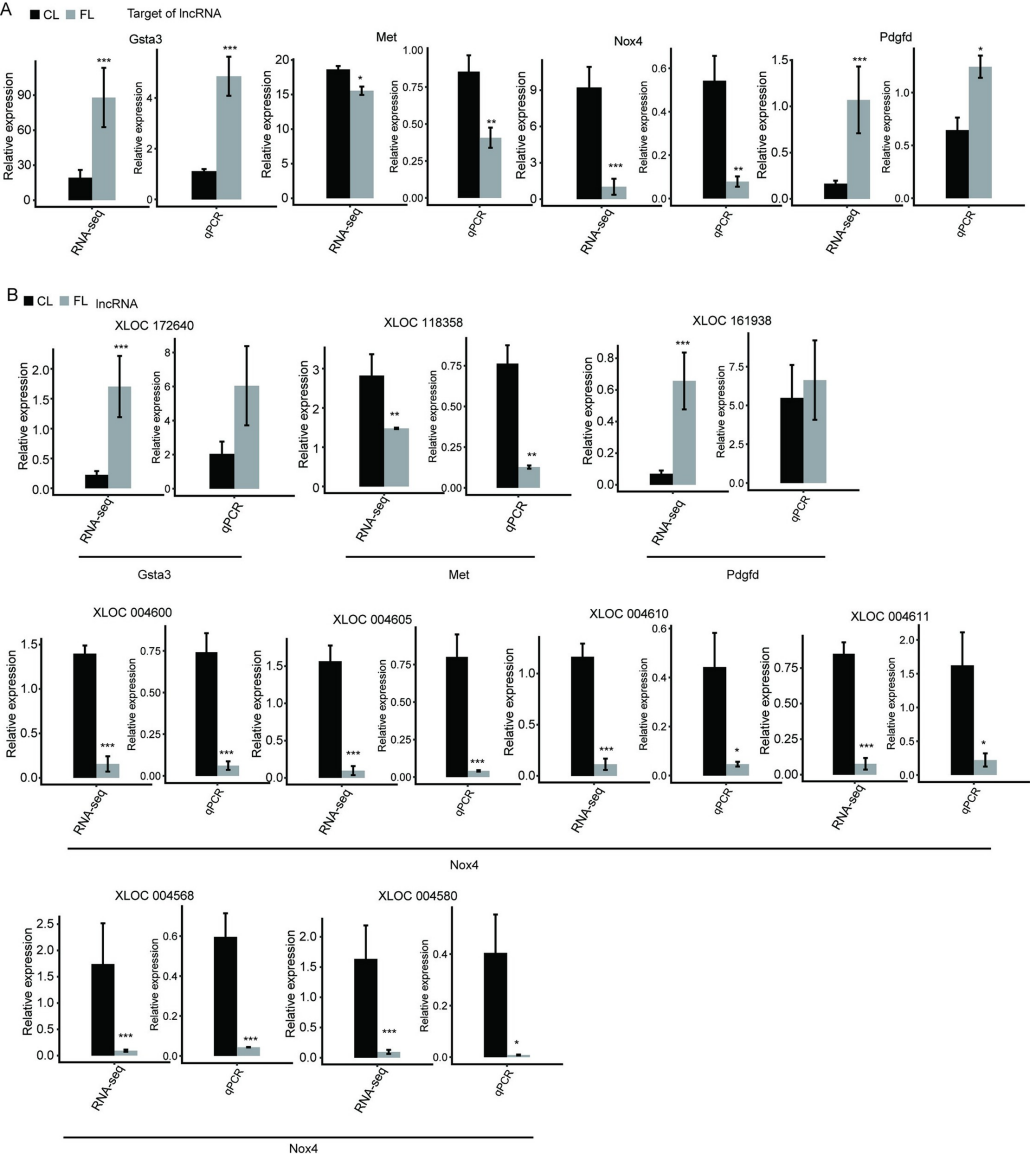
Novel lncRNAs and their targets associated with hepatic fibrogenesis. (A) Expression level of potential cis-acting targets quantified by RNA sequencing analysis and qRT-PCR. Error bars represent mean ± SEM. ***p < 0.001. **p < 0.01. *p < 0.05. (B) Expression level of lncRNAs associated with hepatic fibrogenesis quantified by RNA sequencing analysis and qRT-PCR. Error bars represent mean ± SEM. ***p < 0.001. **p < 0.01. *p < 0.05.

**Fig 3:**
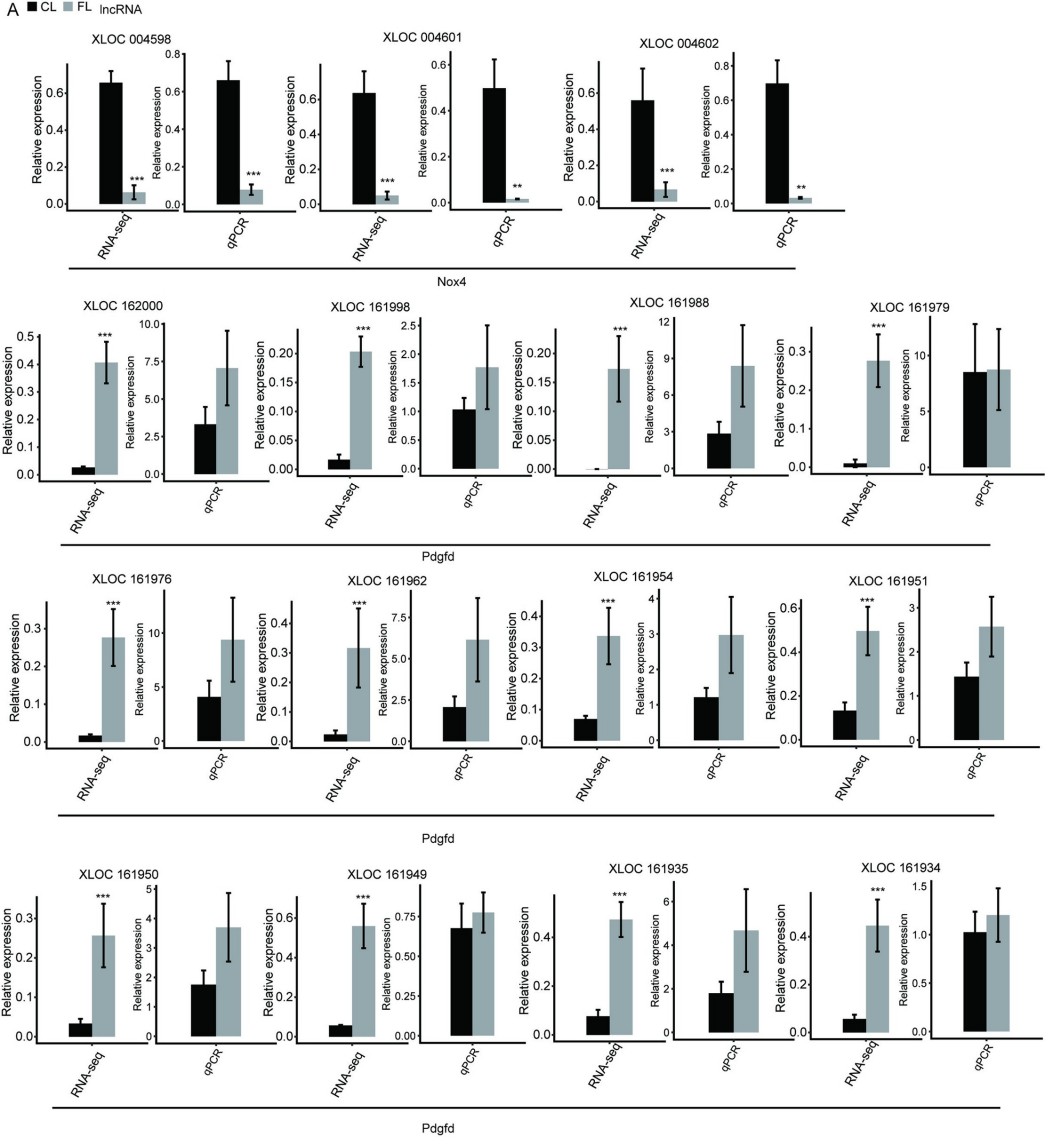
Expression level of lncRNAs associated with hepatic fibrogenesis quantified by RNA sequencing analysis and qRT-PCR. Error bars represent mean ± SEM. *** *p* < 0.001, *p < 0.05.

Our study resulted in a total of 24 DE-lncRNAs among the predicted DE-lncRNAs, according to the selection threshold (fold change ≥2 and p ≤ 0.05) (Figs 2B and 3). Moreover, four potential cis-acting targets (Fig 2A) associated with hepatic fibrosis were identified as labeled by Gsta3, Met, Nox4, and Pdgfd, and their reliability was confirmed by validation experiments. Finally, a total of 10 novel DE-lncRNAs were consistent with the predicted results and had high repeatability, namely XLOC118358, XLCO004600, XLOC004605, XLOC004610, XLOC004611, XLOC004568, XLOC004580 XLOC004598, XLOC004601, and XLOC004602, all of which were downregulated in fibrotic liver tissues (Figs 2B and 3).

Most importantly, our study showed that the cis target gene of DE-lncRNA XLOC118358 was probably Met, and the cis target gene of the other nine DE-lncRNAs mentioned above, XLCO004600, XLOC004605, XLOC004610, XLOC004611, XLOC004568, XLOC004580 XLOC004598, XLOC004601, and XLOC004602, was Nox4. MET, also known as C-MET, is a proto-oncogene that encodes a transmembrane receptor protein and is a member of the receptor tyrosine kinase family. The binding of the MET receptor to its ligand, hepatocyte growth factor (HGF), induces MET dimerization and causes it to enter an activated state, which in turn phosphorylates its substrates to activate downstream signaling pathways. *Nox4* is the gene that encodes reduced nicotinamide adenine dinucleotide phosphooxidase 4, a key enzyme that catalyzes the production of reactive oxygen species (ROS) and plays an important role in a variety of disease processes. Co-expression between DE-lncRNAs and target genes may play a significant role in rats with hepatic fibrosis induced by CCl4.

### Expression and functional annotation of potential cis-acting targets of fibrogenesis-regulated lncRNAs

**Fig 4:**
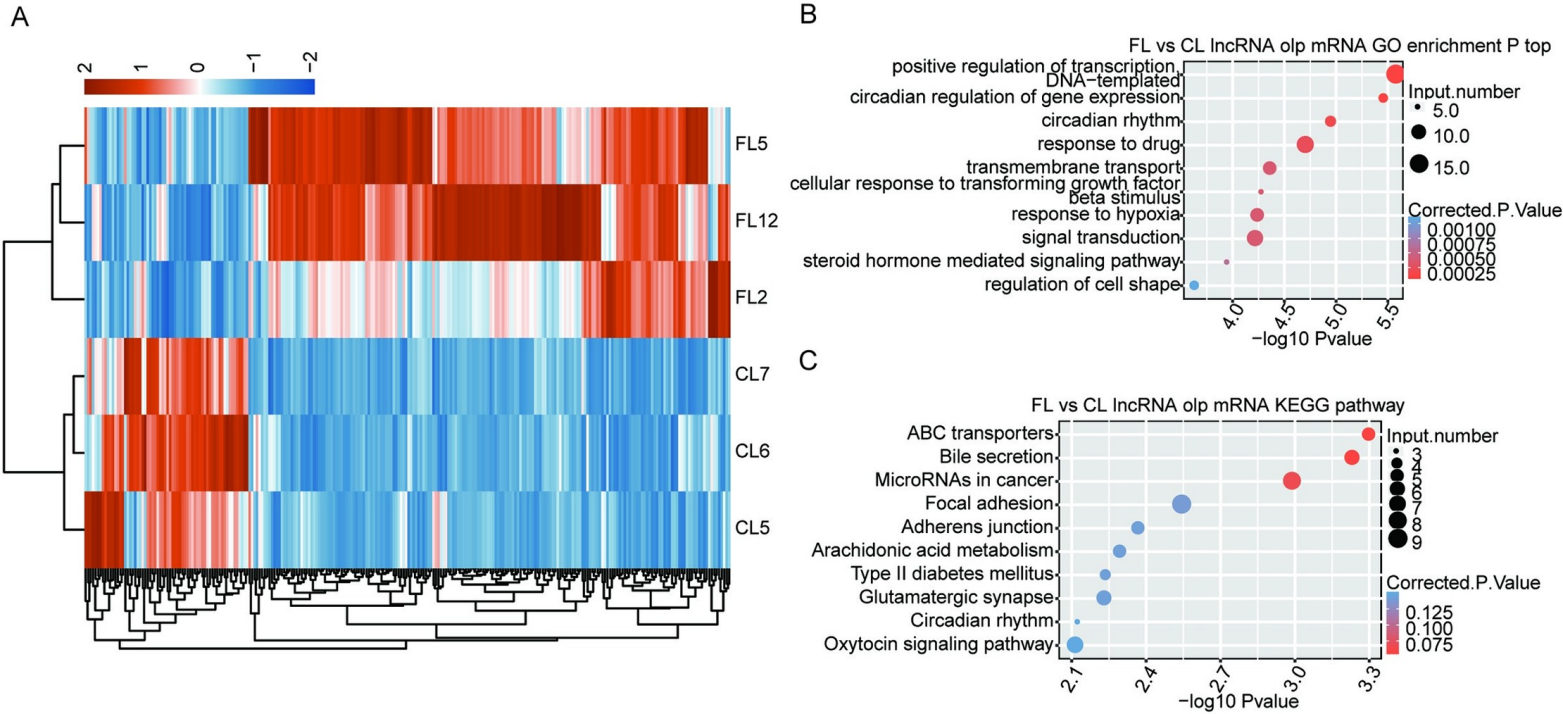
Expression and functional annotation of potential cis-acting targets of fibrogenesis- regulated lncRNAs. (A) Hierarchical clustering of potential cis-acting targets of fibrogenesis- regulated lncRNAs. The color scale represents the median-centered log2 RPKM values. (B) The top 10 enriched GO biological processes of the potential cis-acting targets. (C) The top 10 enriched KEGG pathways of the potential cis-acting targets.

Cis-acting targets are likely to be the target genes regulated by lncRNAs; therefore, we studied the potential function of DE-lncRNAs by functional annotation of cis-genes. For each DE-lncRNA, we obtained expressed genes from its upstream and downstream regions within 10,000 bases, and these genes were overlapped with lncRNA-co-expressed genes to obtain lncRNA targets. The heat map of cis-target hierarchical clustering showed differential expression between the control group and fibrotic liver tissues (Fig 4A). GO functional enrichment analysis and KEGG analysis were involved to identify the biological processes of DE-lncRNAs target genes. The top 10 enriched GO biological processes of the potential cis-acting targets (Fig 4B) were positive regulation of DNA-templated transcription, circadian regulation of gene expression, circadian rhythm, response to drugs, transmembrane transport, cellular response to transforming growth factor β stimulus, response to hypoxia, signal transduction, steroid hormone mediated signaling pathway, and regulation of cell shape.

The top 10 enriched KEGG pathways of the potential cis-acting targets (Fig 4C) were ABC transporters, bile secretion, microRNAs in cancer, focal adhesion, adherens junction, arachidonic acid metabolism, type II diabetes mellitus, glutamatergic synapse, circadian rhythm, and oxytocin signaling pathway.

### DEGs quantified by RNA sequencing analysis and qRT-PCR

**Fig 5:**
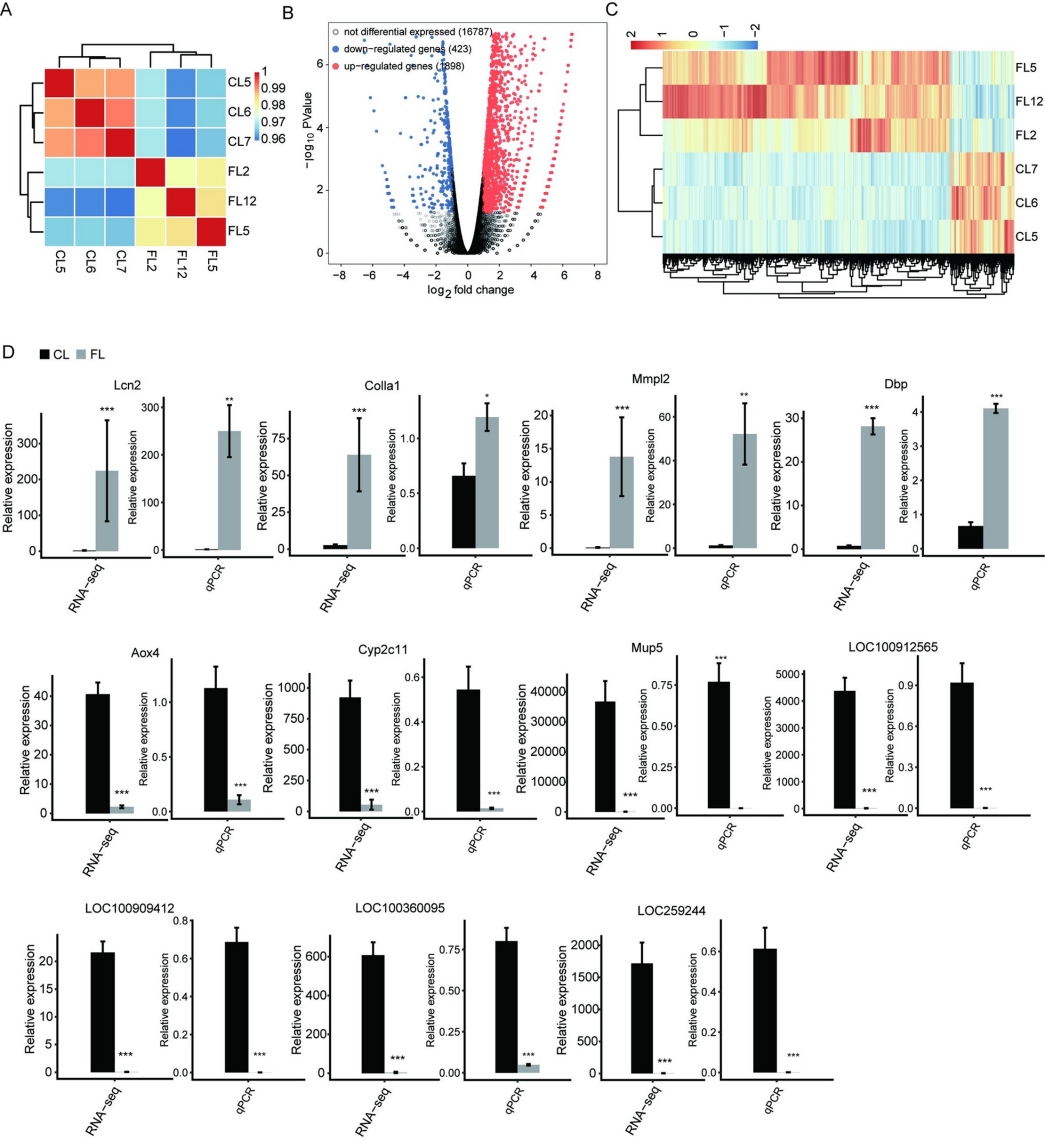
Gene expression alteration in fibrotic rat liver quantified using RNA sequencing analysis and qRT-PCR. (A) Heat map shows the hierarchically clustered Pearson correlation matrix obtained from comparing the transcript expression values for control and fibrotic rat liver samples. (B) Identification of DEGs. Upregulated genes are labeled in red, whereas downregulated are labeled in blue in the volcano plot. FC ≥ 2 or ≤ 0.5, FDR < 0.05. (C) Hierarchical clustering of DEGs in control and fibrotic rat liver samples. The color scale represents the median-centered log2 FPKM values. (D) Differential expression of hepatic fibrogenesis-associated genes. Gene expression were quantified using RNA sequencing data and qRT-PCR. Error bars represent mean ± SEM. ***p < 0.001. **p < 0.01. *p < 0.05.

To investigate possible lncRNA-mRNA interactions, we performed RNA-Seq analysis of mRNA data from control and fibrotic rat liver samples. The heat map shows a significant difference between the two groups (Fig 5A). The results showed that 1,898 genes were upregulated and 423 genes were downregulated in fibrotic liver tissues (Fig 5B), and these gene expressions were well clustered in the two groups of samples (Fig 5C). We included 11 most significant DEGs related to hepatic fibrosis through the selected threshold (FC ≥ 2 or ≤ 0.5, FDR < 0.05) (Fig 5D), and the results of the qPCR validation experiment were consistent with the RNA-seq results. The genes labeled as *Lcn2*, *ColIα1*, *MMp12*, and *Dbp* were upregulated, while *Aox4*, *Cyp2c11*, *Mup5*, *LOC100912565*, *LOC100909412*, *LOC100360095*, and *LOC259244* were downregulated in the process of liver fibrosis.

**Fig 6:**
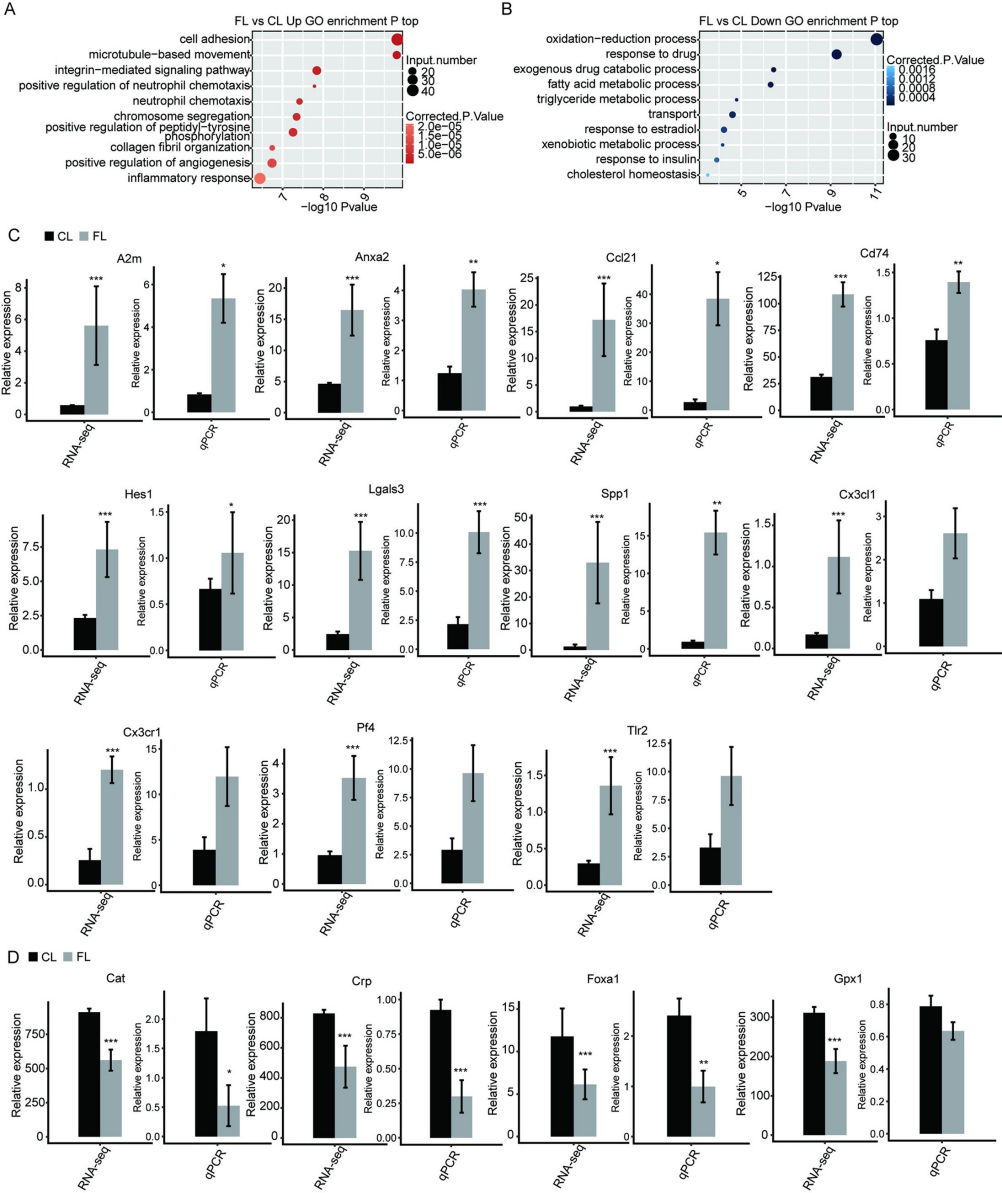
Functional annotation and expression of genes deregulated during hepatic fibrogenesis. (A- B) The top 10 representative GO biological processes of upregulated (A) and downregulated (B) genes. (C-D) Upregulated (C) or downregulated (D) genes expression quantified using RNA sequencing analysis and qRT-PCR. FPKM values were calculated as explained in Materials and Methods. Error bars represent mean ± SEM. ***p < 0.001. **p < 0.01. *p < 0.05.

In addition, we included the top 10 representativly upregulated (Fig 6A) and downregulated (Fig 6B) biological processes of GO terms. We extracted 15 DEGs in the above GO terms and verified the expression of upregulated (Fig 6C) and downregulated (Fig 6D) genes using RNA sequencing analysis and qRT-PCR. Finally, we identified 10 genes deregulated in hepatic fibrosis tissues: genes *A2M*, *Anxa2*, *Ccl21*, *Cd74*, *Hes1*, *Lgals3*, and *Spp1* were upregulated, whereas *Cat*, *Crp*, and *Foxa1* were downregulated in fibrotic liver tissues (Fig 6C and 6D).

### Co-expression network between pathway and DEGs

The top10 GO pathway terms/top 10 KEGG terms and corresponding genes were extracted from DEGs, and the network was drawn with the software Cytoscape (version 3.7.2) to show the relationship between genes and pathways. The processes and metabolic pathways related to liver fibrosis include inhibition of liver repair and detoxification, reduction of liver metabolism, enhancement of liver inflammatory response, promotion of HCS cell activation, promotion of cell cycle and proliferation of HCS, promotion of collagen production and deposition, and enhancement of liver inflammation. Genes *Met* and *Nox4*, the target genes of DE-lncRNAs, were involved in the oxidation-reduction process and PI3K/Akt signaling pathway. The PI3K/Akt signaling pathway was the most enriched signal transduction pathway (S2 and S3 Figs).

## Discussion

We identified 10 novel DE-lncRNAs that are downregulated in the hepatic fibrosis process. The cis target gene of DE-lncRNA XLOC118358 was found to be *Met*, while the cis target gene of the other nine DE-lncRNAs, XLCO004600, XLOC004605, XLOC004610, XLOC004611, XLOC004568, XLOC004580 XLOC004598, XLOC004601, and XLOC004602 was *Nox4*. Regulation of co-expression of lncRNA-Met and lncRNAs-Nox4 is a core mechanism in the hepatic fibrosis process, which can enhance signal factors such as glutathione S-transferase α 3 [36], transforming growth factor-β 1 (TGF—β 1) [5] and platelet-derived growth factor D [37], by activating target genes and decreasing NADPH oxidase 4 [38]. In this study, nine lncRNAs acted together on the same target gene (*Nox4*), indicating the possibility of a cascade including different lncRNAs, which may be a new way to study lncRNAs. Previous research has also shown that different lncRNAs may mediate the same pathway, such as multiple lncRNA-mediated Wnt/β-catenin pathways, regulating the self-renewal of liver cancer stem cells [39]. In nonalcoholic fatty liver disease, lnc-H19 and lnc-Malat1 jointly mediate the stability of SREBP1c [40]. Overall, lncRNAs can activate and regulate the signal transduction pathway and promote the formation of liver fibrosis. In recent decades, several studies have confirmed that lncRNAs can participate in epigenetic regulation of gene expression by participating in important genetic and physiological processes, such as mRNA translation, splicing, protein degradation and phosphorylation modification, subcellular localization of RNA binding protein, and cytoskeleton composition [41–44].

Several previous studies have reported about 5,000 lncRNAs from rat liver tissues. Our study identified several novel lncRNAs related to liver fibrosis by comparison to those reported in a set of 5,000 rat liver-expressed lncRNAs described earlier, and which of the fibrosis-related lncRNAs described in the present study corresponds to one of the 5,000 lncRNAs. By analyzing a rat model of hepatic fibrosis induced by exposure to exogenous chemicals, we identified a number of lncRNAs that are related to hepatic fibrosis, suggesting that exposure to environmental chemicals alters the expression of epigenetic regulators, including xenochemical-responsive lncRNA genes. Kritika et al. [45] reported that rat liver xeno-lnc, rlnc397, which is induced by several nuclear receptor agonists and by the hepatotoxin carbon tetrachloride, shares 66% transcript-transcript identity (TTI) with mouse lncRNA Snhg14. However, the lncRNAs reported in our study share less than 30% TTI with Snhg14, which is overexpressed in several cancers and potentiates tumor progression, in humans, by serving as a sponge (endogenous competitor RNA) for multiple microRNAs [46–48].

In our study, 21 DEGs were screened from the liver tissues of rats with liver fibrosis. Among them, liver fibrosis-related genes *Lcn2*, *Col1α1*, *Mmp12*, *Dbp*, *A2M*, *anxa2*, *Ccl21*, *Cd74*, *Hes1*, *Lgals3*, and *Spp1* were overexpressed in liver fibrosis tissues. Functional analysis of GO and KEGG showed that, in the context of CCl4-induced liver fibrosis, the expression of these genes promoted the inflammatory reaction process of hepatocytes, accelerated the apoptosis of hepatocytes, activated and proliferated HSCs, increased the synthesis of collagen, and promoted the process of liver fibrosis. The downregulated genes in liver fibrosis tissues were *Aox4*, *CYP2C11*, *Mup5*, *Loc100912565*, *Loc100909412*, *Loc100360095*, *Loc259244*, *Cat*, *Crp*, and *Foxa1*. The inhibition of the expression of these genes downregulated the redox process of the liver, decreased the metabolism of exogenous drugs and lipid metabolism including cholesterol and triglycerides, inhibited the normal repair ability of the liver, and promoted the formation of liver fibrosis.

The construction of a pathway-DEG co-expression network showed that lncRNA-Met and lncRNAs-Nox4 may be involved in oxidation-reduction processes and the PI3K/Akt signaling pathway. Furthermore, the PI3K/Akt signaling pathway is the most abundant signal transduction pathway, and it is also one of the most mature pathways and the core of upregulated gene regulation related to liver fibrosis. Through this pathway, TGF β 1, PDGF, Met3, and NOX4 play an important role in the process of liver fibrosis [49–51]. PI3K is a heterodimer with dual activities of phosphatidylinositol kinase and serine/threonine protein kinase (Akt). Akt is an important target kinase downstream of PI3K with serine and threonine kinase activities [52]. Upregulation of lncRNAs results in the overexpression of downstream target genes, such as *TGF β 1*, *PDGF*, and *Met3*. The increase in signal factors secreted by HSCs, such as transforming growth factor-β1 [53], glutathione S-transferase, platelet-derived growth factor [54] and insulin-like growth factors [55], activates PI3K, which catalyzes the phosphorylation of phosphatidylinositol 4,5-diphosphate (PIP2) to phosphatidylinositol 3,4,5-triphosphate (PIP3), and then activates the downstream target protein kinase B (PKB) of PIP3, namely Akt [56,57]. Activated Akt can initiate cascade reactions in HSCs, activate HSCs, and promote the synthesis of ECM, leading to liver fibrosis [58]. At present, several lncRNAs have been proved to be involved in the occurrence and development of liver fibrosis by controlling PI3K/Akt signaling pathway, such as lincrna-p21 [59], HOTAIR [60], and H19 [20,21].

## Conclusion

We identified 10 novel lncRNAs associated with liver fibrosis, with *Met* and *Nox4* as their target genes. Co-expression between lncRNA-Met and lncRNAs-Nox4 may activate HSCs through oxidation-reduction processes and PI3K/Akt signaling pathway, increase the production of ECM, and regulate the process of liver fibrosis. The discovery of novel lncRNAs associated with liver fibrosis may provide new targets for the treatment of liver fibrosis. There are some limitations in this study, while the authors identified lncRNAs involved in fibrosis changes by RNAs involved in the total chunk of liver that were induced by CCl4. Further research in particular cell types is needed to understand the specificity of lncRNAs in different tissues. Furthermore, assessing the differential expression of lncRNAs in different cell locations (nucleus or cytoplasm) is helpful for studying the function of lncRNAs.

## Supporting information

S1 Fig Establishment of CCl4-induced hepatic fibrosis in rats. (TIF)

S2 Fig Co-expression network between pathway and DEGs. (TIF)

S3 Fig Cytoscape networks for *Met* and *Nox4*. (TIF)

S4 Fig Genome browser screen for *Met*. (TIF)

S5 Fig Genome browser screen for *Nox4*. (TIF)

S1 Table FPKM of DEGs & DElncRNAs. (XLSX)

S1 Appendix GTF files with gene structures and all isoform structures for lncRNAs. (ZIP)

S2 Appendix Table for the main Results text listing all regulated lncRNAs. (ZIP)
