# Supplementary figures and images for: Identification of differentially expressed long noncoding RNAs and pathways in liver tissues from rats with hepatic fibrosis
Source: PLoS One. 2021 Oct 1;16(10):e0258194. doi: 10.1371/journal.pone.0258194 (PMC8486097; doi:10.1371/journal.pone.0258194)

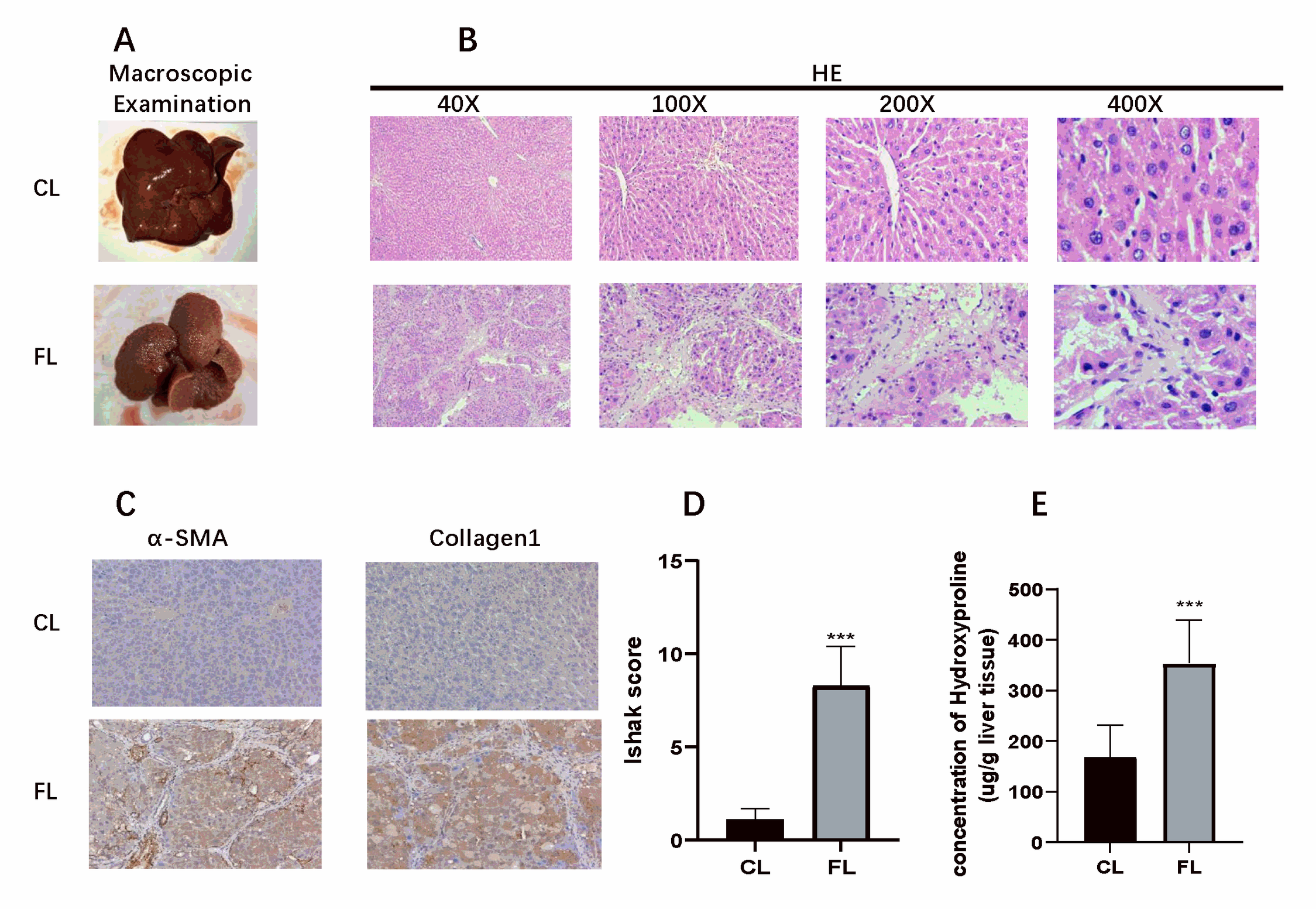

Supplement: S1 Fig — (TIF) [file pone.0258194.s001.tif]

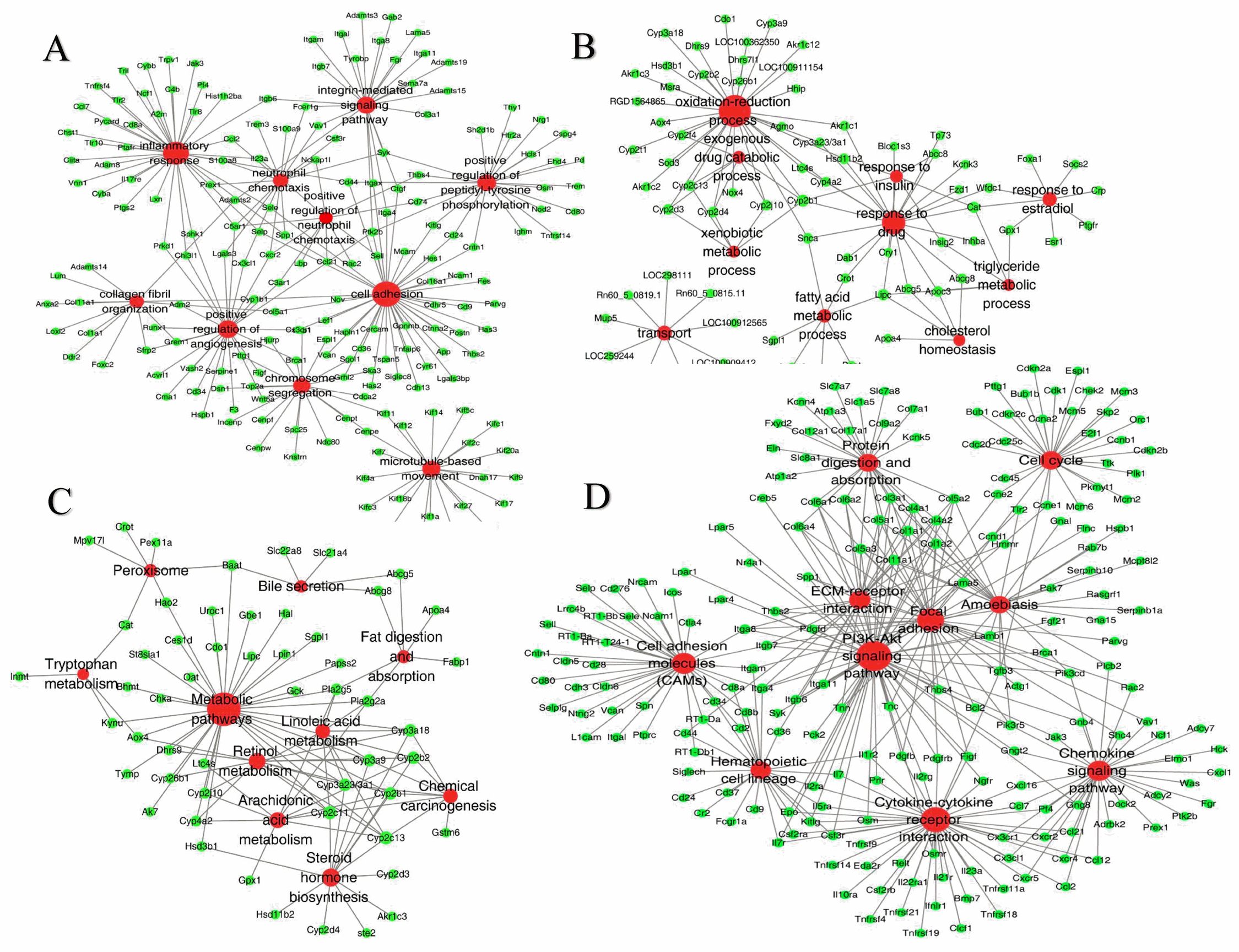

Supplement: S2 Fig — (TIF) [file pone.0258194.s002.tif]

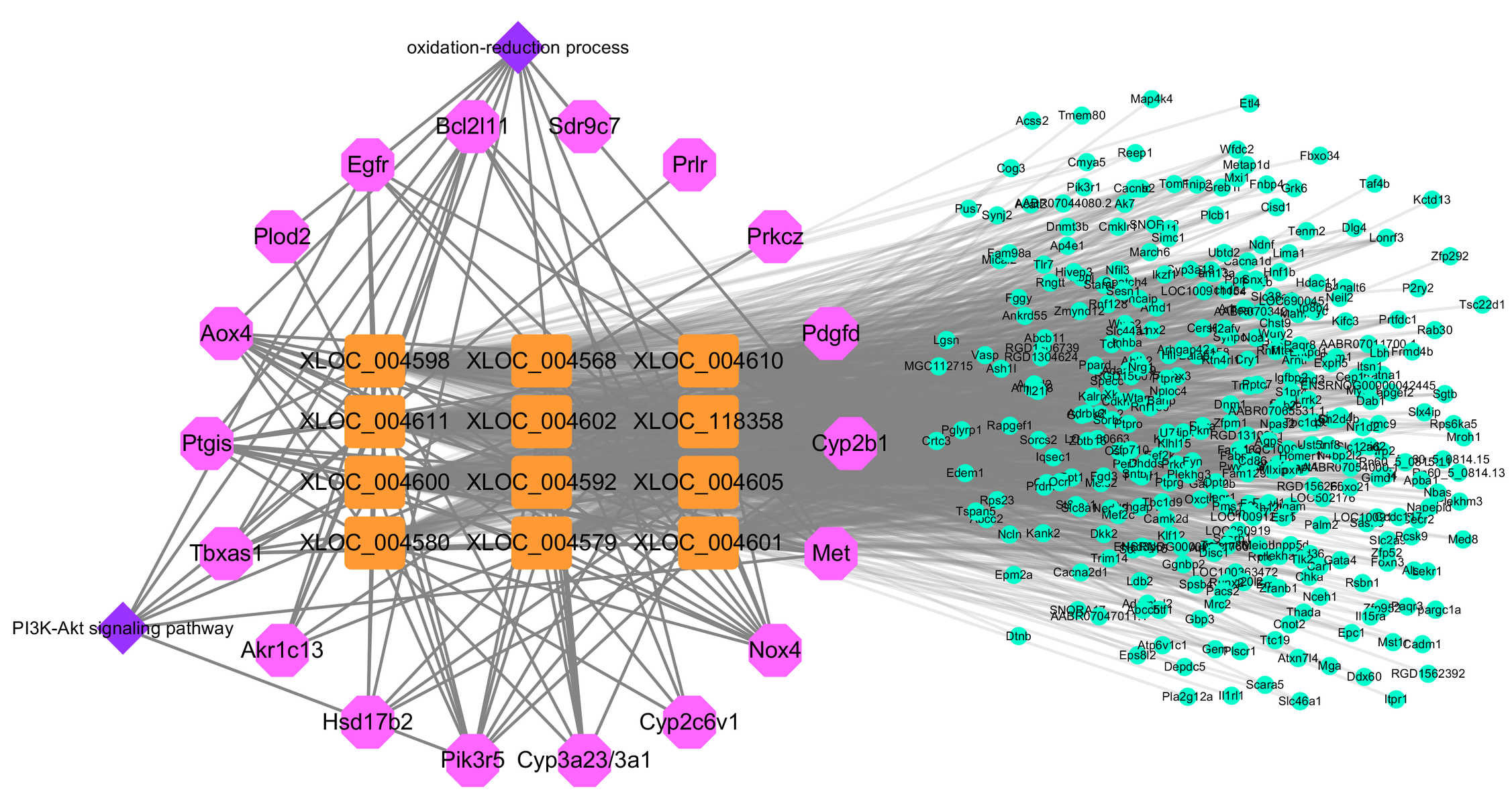

Supplement: S3 Fig — (TIF) [file pone.0258194.s003.tif]

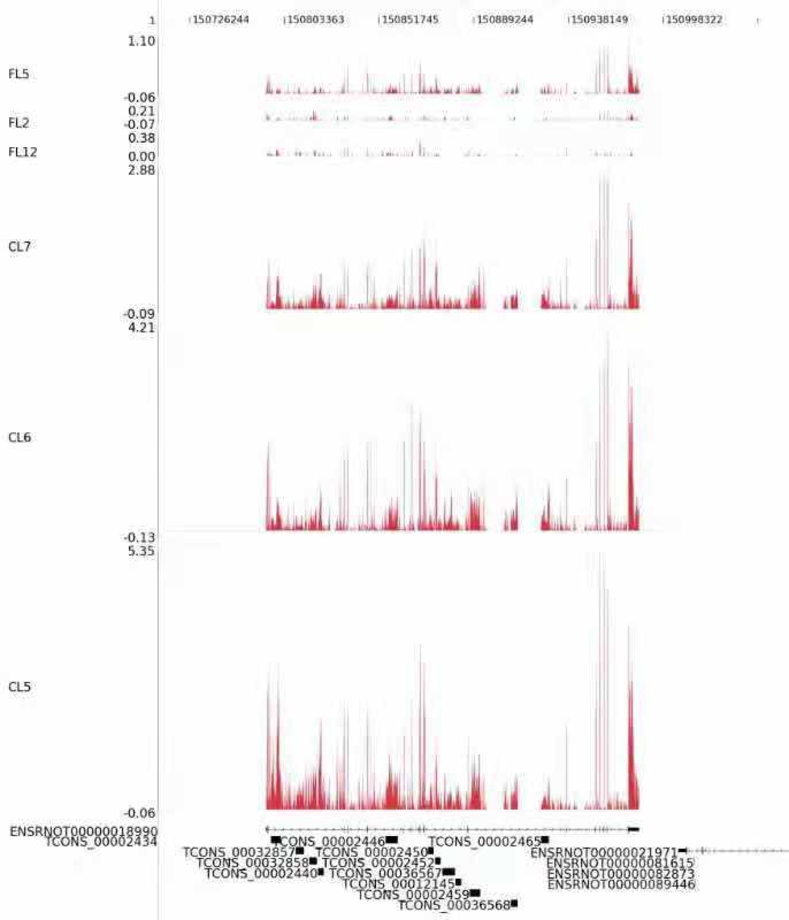

Supplement: S4 Fig — (TIF) [file pone.0258194.s004.tif]

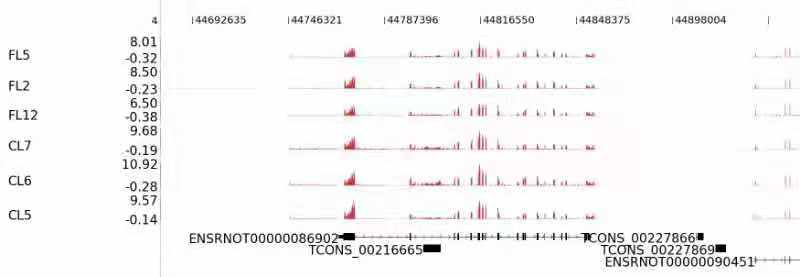

Supplement: S5 Fig — (TIF) [file pone.0258194.s005.tif]
